# Exploring Causal Relationships in Mental Health Literacy Through Twitter Content: A Machine Learning Approach

**DOI:** 10.1192/j.eurpsy.2024.268

**Published:** 2024-08-27

**Authors:** Y.-J. Lien, H.-P. Feng, C.-H. Chen, Y.-H. Tseng, W.-H. Tseng

**Affiliations:** ^1^Department of Health Promotion and Health Education; ^2^Graduate Institute of Library & Information Studies, National Taiwan Normal University, Taipei, Taiwan, Province of China

## Abstract

**Introduction:**

The concept of Mental Health Literacy (MHL) is inherently multidimensional. However, the interrelationships among its various dimensions remain insufficiently elucidated. In recent years, the textual analysis of social media posts has emerged as a promising methodological approach for longitudinal research in this domain.

**Objectives:**

This study aimed to investigate whether temporal causal associations exist between recognition of mental illness (R), mental illness stigma (S), help-seeking efficacy (HE), maintenance of positive mental health (M), and help-seeking attitude (HA).

**Methods:**

Tweets were collocted at three distinct time points: T1, T2, and T3, spanning the period from November 1, 2021, to December 31, 2022. We employed a machine-learning approach to categorize the posts into five MHL facets. Using these facets, we trained a machine learning model, specifically Bidirectional Encoder Representations from Transformers (BERT), to determine the MHL scores. To be eligible, an account must have an R facet score at T1, and M, S, HE facet scores at T2, as well as an HA facet score at T3. In total, we retrieved 4,471,951 MHL-related tweets from 941 users. We further employed structural equation modeling to validate the causal relationships within the MHL framework.

**Results:**

In the evaluation, BERT achieved average accuracy scores exceeding 89% across the five MHL facets in the validation set, along with F1-scores ranging between 0.75 and 0.89. Among the five MHL facets—maintenance of positive mental health, recognition of mental illness, help-seeking efficacy, and help-seeking attitudes—each demonstrated a statistically significant positive correlation with the others. Conversely, mental illness stigma exhibited a statistically significant negative correlation with the remaining four facets. In the analysis using single-mediation models, each of the individual mediator variables—namely, mental illness stigma, help-seeking efficacy, and maintenance of positive mental health—exhibited significant indirect effects. In the multiple-mediation model, two mediator variables—help-seeking efficacy and maintenance of positive mental health—demonstrated significant indirect effects. These findings suggested that the recognition of mental illness exerted an influence on help-seeking attitudes through one or more of these mediators.

**Conclusions:**

By leveraging machine learning techniques for the textual analysis of social media and employing a longitudinal research design with panel data, this study elucidates the potential mechanisms through which the MHL framework influences attitudes toward seeking mental health services. These insights hold significant implications for the design of future interventions and the development of targeted policies aimed at promoting help-seeking behaviors.

**Disclosure of Interest:**

None Declared

